# Prognostic Nutritional Index and Lung Immune Prognostic Index as Prognostic Predictors for Combination Therapies of Immune Checkpoint Inhibitors and Cytotoxic Anticancer Chemotherapy for Patients with Advanced Non-Small Cell Lung Cancer

**DOI:** 10.3390/diagnostics12020423

**Published:** 2022-02-06

**Authors:** Satomi Tanaka, Junji Uchino, Takashi Yokoi, Takashi Kijima, Yasuhiro Goto, Yoshifumi Suga, Yuki Katayama, Ryota Nakamura, Kenji Morimoto, Akira Nakao, Makoto Hibino, Nozomi Tani, Takayuki Takeda, Hiroyuki Yamaguchi, Yusuke Tachibana, Chieko Takumi, Noriya Hiraoka, Masafumi Takeshita, Keisuke Onoi, Yusuke Chihara, Ryusuke Taniguchi, Takahiro Yamada, Yohei Matsui, Osamu Hiranuma, Yoshie Morimoto, Masahiro Iwasaku, Shinsaku Tokuda, Yoshiko Kaneko, Tadaaki Yamada, Koichi Takayama

**Affiliations:** 1 Department of Pulmonary Medicine, Graduate School of Medical Science, Kyoto Prefectural University of Medicine, 465 Kajii-cho Kawaramachi-hirokoji, Kamigyo-ku, Kyoto 602-8566, Japan; satanaka@koto.kpu-m.ac.jp (S.T.); suga4423@koto.kpu-m.ac.jp (Y.S.); ktym2487@koto.kpu-m.ac.jp (Y.K.); ryotan@koto.kpu-m.ac.jp (R.N.); m-kenji@koto.kpu-m.ac.jp (K.M.); yoshie-m@koto.kpu-m.ac.jp (Y.M.); miwasaku@koto.kpu-m.ac.jp (M.I.); tokku@koto.kpu-m.ac.jp (S.T.); kaneko-y@koto.kpu-m.ac.jp (Y.K.); tayamada@koto.kpu-m.ac.jp (T.Y.); takayama@koto.kpu-m.ac.jp (K.T.); 2 Department of Respiratory Medicine and Hematology and Department of Thoracic Oncology, Hyogo College of Medicine, 1-1 Mukogawachō, Nishinomiya 663-8501, Japan; ta-yokoi@hyo-med.ac.jp (T.Y.); tkijima@hyo-med.ac.jp (T.K.); 3 Department of Respiratory Medicine, Fujita Health University, 1-98 Dengakugakubo, Kutsukake-cho, Toyoake 470-1192, Japan; gotoyasu510@gmail.com; 4 Department of Respiratory Medicine, Faculty of Medicine, Fukuoka University, 8 Chome-19-1 Nanakuma, Jonan Ward, Fukuoka 814-0180, Japan; akiran@fukuoka-u.ac.jp; 5 Department of Respiratory Medicine, Shonan Fujisawa Tokushukai Hospital, 1 Chome-5-1 Tsujidokandai, Fujisawa 251-0041, Japan; makotohibino560328@yahoo.co.jp; 6 Department of Respiratory Medicine, Japanese Red Cross Kyoto Daini Hospital, 355-5, Haruobicho, Kamaza Dori Marutamachi Agaru, Kamigyo Ward, Kyoto 602-8026, Japan; nozomi-t@koto.kpu-m.ac.jp (N.T.); dyckw344@yahoo.co.jp (T.T.); 7 Department of Respiratory Medicine, Nagasaki University Graduate School of Biomedical Sciences, 1-7-1 Sakamoto, Nagasaki 852-8501, Japan; yamaguchi-hiroyuki@umin.ac.jp; 8 Department of Respiratory Medicine, Japanese Red Cross Kyoto Daiichi Hospital, 15-749 Higashiyama Ward, Honmachi, Kyoto 605-0981, Japan; yutachib@koto.kpu-m.ac.jp (Y.T.); chieko-takumi@kyoto1-jrc.org (C.T.); noriya-hiraoka@kyoto1-jrc.org (N.H.); 9 Department of Respiratory Medicine, Ichinomiya Nishi Hospital, Hira-1 Kaimei, Ichinomiya City 494-0001, Japan; takefumi0415@outlook.jp; 10 Uji-Tokushukai Medical Center, Department of Respiratory Medicine, 145 Ishibashi Makishimacho, Uji-shi 611-0041, Japan; onoi@koto.kpu-m.ac.jp (K.O.); c1981311@koto.kpu-m.ac.jp (Y.C.); 11 Department of Pulmonary Medicine, Matsushita Memorial Hospital, 5-55 Sotojima-cho, Moriguchi 570-8540, Japan; ryusuketaniguchi3@gmail.com (R.T.); yamada.takahiro007@jp.panasonic.com (T.Y.); 12 Department of Pulmonary Medicine, Otsu City Hospital, 2-9-9 Motomiya, Otsu-City 520-0804, Japan; y-matsui@koto.kpu-m.ac.jp (Y.M.); osamu319@true.ocn.ne.jp (O.H.)

**Keywords:** chemoimmunotherapy, advanced non-small cell lung cancer, prognostic nutritional index, lung immune prognostic index, programmed cell death ligand 1

## Abstract

Combination therapy with immune checkpoint inhibitors and cytotoxic chemotherapies (chemoimmunotherapy) is associated with significantly better survival outcomes than cytotoxic chemotherapies alone in patients with advanced non-small cell lung cancer (NSCLC). However, there are no prognostic markers for chemoimmunotherapy. The prognostic nutritional index (PNI) and lung immune prognostic index (LIPI) are prognostic biomarkers for immune checkpoint inhibitor (ICI) monotherapy or cytotoxic chemotherapies. Thus, we aimed to examine whether these factors could also be prognostic markers for chemoimmunotherapy. We retrospectively examined 237 patients with advanced NSCLC treated with chemoimmunotherapy. In the total group, the median overall survival (OS) was not reached, and the median progression-free survival (PFS) was 8.6 months. Multivariate analysis of OS and PFS revealed significant differences based on PNI and LIPI. Programmed cell death ligand 1 (PD-L1) was also significantly associated with OS and PFS. PNI and a PD-L1 tumor proportion score (TPS) of <50% and poor LIPI (regardless of PD-L1 TPS) were associated with poor prognosis. PNI and LIPI predicted survival outcomes in patients with advanced NSCLC treated with chemoimmunotherapy, especially in patients with PD-L1 TPS <50%. For patients in this poor category, chemoimmunotherapy may result in a worse prognosis than expected.

## 1. Introduction

Combination therapy with immune checkpoint inhibitors (ICIs) and chemotherapy (hereafter chemoimmunotherapy) is currently approved and used as the first-line treatment for advanced non-small cell lung cancer (NSCLC). Chemoimmunotherapy regimens that can be used for advanced NSCLC include pembrolizumab (Pemb) plus a platinum-based drug plus pemetrexed (PEM) (as per the KEYNOTE-189 trial) [1], Pemb plus carboplatin (CBDCA) plus paclitaxel (PTX) or nab-PTX (as per the KEYNOTE-407 trial) [2], atezolizumab (Atezo) plus CBDCA plus PTX plus bevacizumab [3], and Atezo plus a platinum-based drug plus PEM [4]. All these regimens have shown significant improvements in progression-free survival (PFS) and safety when compared to regimens of chemotherapy alone.

An additional report from the KEYNOTE-189 trial has stated that the median overall survival (OS) in the chemoimmunotherapy group was 22.0 months, which was more than twice the median OS of 10.7 months in the chemotherapy alone group [5]. Thus, with the advent of each new regimen, the prognosis of patients with advanced NSCLC has significantly improved. However, in the aforementioned studies, the Kaplan–Meier curve for OS declined just after the introduction of treatment, and in fact, some patients did not respond to the combination therapy, although they seemed to maintain good performance status in clinical practice. Therefore, the establishment of a prognostic marker is required to create a more appropriate treatment selection.

For monotherapy with ICIs, treatment selection is based on the programmed cell death ligand 1 (PD-L1) tumor proportion score (TPS); however, this score does not necessarily correlate with therapeutic effect or prognosis. The predictors for monotherapy have not yet been established. However, various prognostic markers and predictive markers of more than 20 factors are currently being investigated using pretreatment clinical data because of early approval [6,7]. The prognostic nutritional index (PNI), which is one of the most well-established markers, is an index first proposed by Onodera et al. in the 1980s as an estimate of the prognosis of the postoperative course of patients with gastrointestinal cancer [8]. Studies have compared PNI before and after surgery to establish its usefulness in predicting the prognosis of patients before treatment with anticancer drugs [9]. It can also be a biomarker for predicting the therapeutic effect, even in ICI monotherapy [10]. PNI is calculated as 10 × albumin (Alb) (g/dL) + 0.005 × lymphocyte count (/μL), and it can be easily evaluated in the clinical setting. Onodera et al. set PNI 40 as the cut-off value; however, a standard value has not yet been determined, and there are also reports that adjustments according to age may be necessary [11].

The lung immune prognostic index (LIPI) is a scoring system based on lactate dehydrogenase (LDH) level and the neutrophil-to-lymphocyte ratio (NLR), which was proposed by Mezquita et al. as a new lung immune index in 2018 [12]. LIPI is considered to be a biomarker for predicting the prognosis of patients treated with ICI monotherapies, including Pemb and Atezo [12,13]. The LIPI can also predict the prognosis of patients who receive cytotoxic anticancer drugs and epidermal growth factor receptor (EGFR)-tyrosine kinase inhibitor treatments [14]. Furthermore, the LIPI is based on an NLR > 3 and an LDH level greater than the upper limit of normal (ULN). It is divided into three ratings: good, zero factors; intermediate, one factor; and poor, two factors. The ULN for LDH is defined according to the limits determined by each hospital. It is a score that is easy to incorporate into daily medical practice because it is evaluated on a scale of 0, 1, or 2. Regarding the cut-off value of NLR, the largest published study of ICIs for patients with cancer [15] determined the value to be 3.

In the current study, we retrospectively analyzed real-world data of chemoimmunotherapy in Japan and examined whether PNI and LIPI could be prognostic markers for chemoimmunotherapy.

## 2. Results

### 2.1. Baseline Patient Characteristics

The baseline characteristics of patients are presented in Table 1.

Among the 253 patients treated with chemoimmunotherapy, 237 were enrolled in the study (Supplementary Figure S1).

The median age was 69 (interquartile range (IQR], 62–73) years, and a majority (78.9%) of the patients were male. The median body mass index (BMI) before treatment was 21.5 (IQR, 19.4–23.3) kg/m^2^, and 57.0% of patients had BMIs <22 kg/m^2^. Regarding the PD-L1 TPS status, 71 (30.0%) patients had a PD-L1 TPS ≥ 50%, 81 (34.2%) had a PD-L1 TPS of 1–49%, and the remaining patients were PD-L1-negative. Eastern Cooperative Oncology Group Scale of Performance Status (ECOG-PS) scores of 0, 1, and 2 were observed in 90 (38.0%), 134 (56.5%), and 10 (4.2%) patients, respectively. Clinical stages IIIB and IIIC/IV, according to the tumor-node-metastasis classification of malignant tumors (eighth edition), were observed in 18 and 174 patients, respectively. Recurrence after definitive treatment (operation or chemoradiation) was noted in the remaining patients (*n* = 45). Regarding chemoimmunotherapy regimens, 124 (52.3%) patients were treated with CBDCA + PEM + Pemb, and 83 (35.0%) patients were treated with CBDCA + PTX/nab-PTX + Pemb. Other patients were treated with an Atezo regimen.

### 2.2. Survival and Response Rate in the Total Group

**Figure 1:**
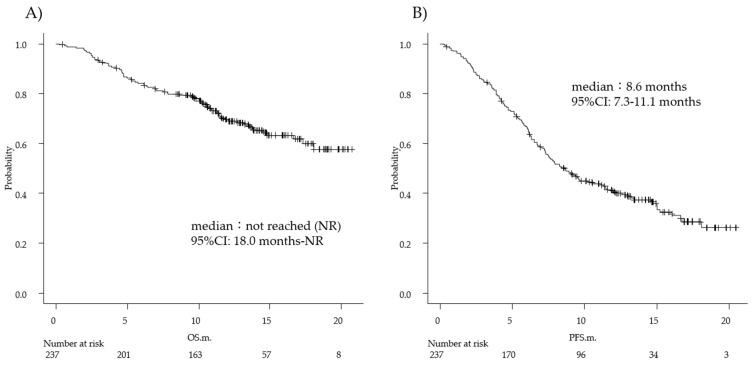
Overall survival (OS) and progression-free survival (PFS) in the overall analysis group. At the time of analysis, 67.1% of patients were alive at a median follow-up time of 11.7 (interquartile range, 8.7–14.9) months. Kaplan–Meier estimates of (**A**) OS and (**B**) PFS in the overall analysis group. Tick marks indicate censoring of data at the last time the patient was known to be alive (**A**) and alive without disease progression (**B**). Median OS was not reached (95% confidence interval, 18.0–not reached). The median PFS was 8.6 (95% confidence interval, 7.3–11.1) months.

The overall response rate (ORR) was 59.1% (complete response, 6.3%; partial response, 52.7%), whereas the disease control rate (DCR) was 87.3%. At the time of analysis, 67.1% of patients were alive, at a median follow-up time of 11.7 (IQR, 8.67–14.9) months. The median OS was not reached, and the median PFS was 8.6 (95% confidence interval [CI], 7.3–11.1) months (Figure 1). Based on this median PFS result, we calculated the PNI cut-off value using a PNI receiver operating characteristic (ROC) curve analysis. The cut-off value was 40.35 (sensitivity, 52.0%; specificity, 74.6%; area under the curve [AUC] of the ROC curve, 0.63 [95% CI, 0.56–0.70]). When NLR was calculated in the same way, the cut-off value was 3.74 (sensitivity, 53.7%; specificity, 64.0%; AUC of the ROC curve, 0.60 [95% CI, 0.53–0.67]), which was higher than the cut-off value set based on LIP (NLR = 3).

In terms of survival, the median OS was not reached in either the PD-L1 TPS < 1% or PD-L1 TPS ≥ 50% group, and it was 18.0 months (95% CI, 14.8–not reached) in the PD-L1 TPS 1–49% group (*p* = 0.51). The median PFS was 7.8 months (95% CI, 6.2–11.3) in the PD-L1 TPS < 1% group, 7.3 months (95% CI, 5.5–11.1) in the PD-L1 TPS 1–49% group, and 11.6 months in the PD-L1 TPS ≥ 50% group (*p* = 0.054) (Supplementary Figure S2).

### 2.3. Univariate and Multivariate Analyses of Overall Survival in the Overall Analysis Group

The results of univariate and multivariate analyses of factors associated with OS are summarized in Table 2.

Univariate and multivariate analyses revealed that age <75 years (hazard ratio [HR], 2.39; 95% CI, 1.29–4.435; *p* = 0.01), a LIPI of 0 or 1 (HR, 2.75; 95% CI, 1.48–5.11; *p* < 0.001), and a PNI ≥ 40.35 (HR, 2.38; 95% CI, 1.23–4.62; *p* = 0.01) were favorable independent prognostic factors. PD-L1 TPS was also a significant factor in the multivariate analysis (HR, 0.53; 95% CI, 0.30–0.95; *p* = 0.03), but it was not significant in the univariate analysis (*p* = 0.30).

### 2.4. Univariate and Multivariate Analyses of Progression-Free Survival in the Total Group

The results of the univariate and multivariate analyses of factors associated with PFS are summarized in Supplementary Table S1. Univariate and multivariate analyses revealed that a LIPI of 0 or 1 (HR, 2.01; 95% CI, 1.28–3.15; *p* < 0.001), a PNI ≥ 40.35 (HR, 1.72; 95% CI, 1.07–2.79; *p* = 0.03), and a PD-L1 TPS ≥ 50% (HR, 0.42; 95% CI, 0.27–0.65; *p* < 0.001) were favorable independent prognostic factors.

### 2.5. Association between the Prognostic Nutritional Index and Clinical Outcomes of Chemoimmunotherapy

**Figure 2:**
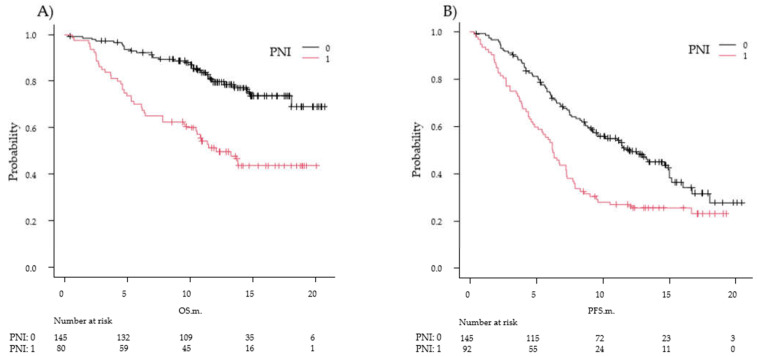
Survival according to the prognostic nutritional index (PNI) in the overall analysis group. Kaplan–Meier estimates of overall survival (OS) (**A**) and progression-free survival (PFS) (**B**) according to the PNI in the overall analysis group. The black line represents the high-PNI group (score 0) with good nutritional status, and the red line represents the low-PNI group (score 1) with poor nutritional status. The two groups differ significantly in both OS and PFS. In (**A**), the median OS was not reached in the PNI 0 group and was 12.1 months in the PNI 1 group (hazard ratio (HR), 3.0; *p* < 0.001). In (**B**), the median PFS was 12.0 months in the PNI 0 group and 6.2 months in the PNI 1 group (HR, 1.9; *p* < 0.001).

Among the 237 patients evaluated, the median PNI was 42.6 (IQR, 37.3–47.9). Of those patients, 145 (61.2%) had a good PNI ≥ 40.35 (score of 0), and 92 (38.8%) had a poor PNI < 40.35 (score of 1). The DCRs were 90.3% and 82.5% in the PNI 0 and PNI 1 groups, respectively (*p* = 0.096). The median OS was not reached in the PNI 0 group and was 12.1 (95% CI, 9.5–not reached) months in the PNI 1 group (*p* < 0.001). The median PFS was 12.0 (95% CI, 9.2–15.0) months in the PNI 0 group and 6.2 (95% CI, 4.9–7.3) months in the PNI 1 group (*p* < 0.001) (Figure 2).

In the PNI 0 group, the median OS was not reached, and there was no statistically significant difference among the three PD-L1 TPS groups (*p* = 0.87). Moreover, in the PNI 1 group with poor nutrition (PD-L1 TPS ≥ 50%), the median survival was not reached, and this was statistically different from that of the PD-L1 TPS < 50% group (*p* = 0.0453). In the PNI 1 group, patients with PD-L1 TPS < 50% had poorer survival compared with the PD-L1 TPS ≥ 50% group (Supplementary Figure S3). A similar trend was observed for PFS. In the PNI 0 group, the median OS was not reached, and there were no significant differences among the PD-L1 TPS group (*p* = 0.76). In the PNI 1 group, the median PFS in the PD-L1 TPS ≥ 50% subgroup was statistically significantly different among the three levels of PD-L1 TPS groups (*p* < 0.001). The median PFS in the PD-L1 TPS ≥50% subgroup was also better than the PFS in the overall analysis group (9.6 months (95% CI, 6.3–not reached) and 8.6 months, respectively; *p* < 0.001) (Supplementary Figure S4).

### 2.6. Association between the Lung Immune Prognostic Index and Clinical Outcomes in the Chemoimmunotherapy Group

**Figure 3:**
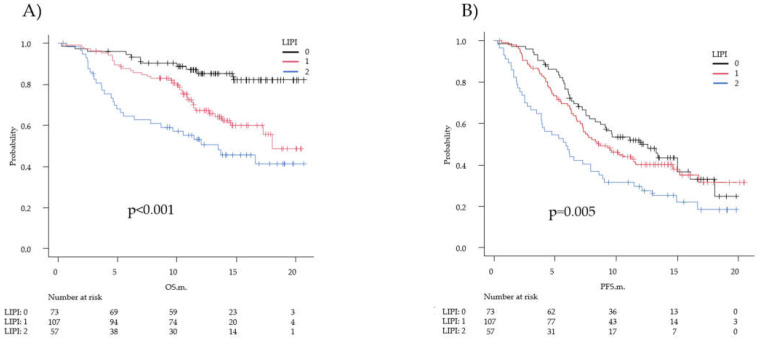
Survival according to the lung immune prognostic index (LIPI) in the overall analysis group. Kaplan–Meier estimates of overall survival (OS) (**A**) and progression-free survival (PFS) (**B**) according to the LIPI in the overall analysis group. The black line represents the LIPI 0 (good immune status) group, the red line represents the LIPI 1 (median immune status) group, and the green line represents the LIPI 2 (poor immune status) group. These groups differed significantly in both OS and PFS. In (**A**), the median OS was not reached in the LIPI 0 group, was 18.0 months in the LIPI 1 group, and was 13.5 months in the LIPI 2 group (*p* < 0.001). In (**B**), the median PFS was 12.6 months in the LIPI 0 group, 8.7 months in the LIPI 1 group, and 5.9 in the LIPI 2 group (*p* = 0.005).

The median dNLR was 3.4 (IQR, 2.44–5.31), and 143 patients (60.3%) had dNLR > 3. Seventy-eight patients had LDH > ULN. Among the 237 patients evaluated for LIPI, 73 (30.8%) had a good LIPI (score of 0), 107 (45.1%) had an intermediate LIPI (score 1), and 57 (24.1%) had a poor LIPI (score 2). The DCRs were 93.2%, 86.9%, and 80.7% in the LIPI 0, 1, and 2 groups, respectively (*p* = 0.101). The median OS was not reached in the LIPI 0 group, was 18.0 (95% CI, 14.5–not reached) months in the LIPI 1 group, and was 13.5 (95% CI, 6.4–not reached) months in the LIPI 2 group (*p* < 0.001). The median PFS was 12.6 (95% CI, 8.3–16.0) months, 8.7 (95% CI, 7.0–11.6) months, and 5.9 (95% CI, 3.9–7.9) months (*p* = 0.005) in the LIPI 0, LIPI 1, and LIPI 2 groups, respectively (Figure 3).

The OS in the LIPI 0, 1, and 2 groups according to PD-L1 TPS were as follows. In the LIPI 0 and LIPI 1 groups (good and intermediate immune groups), the median OS was not reached for any PD-L1 TPS subgroup, except in the PD-L1 TPS 1–49% subgroup of LIPI 1 patients. No statistically significant difference in OS was observed among PD-L1 TPS subgroups (*p* = 0.32 in the LIPI 0 group and *p* = 0.66 in the LIPI 1 group). In the LIPI 2 group (poor immune group), the median survival was not reached in patients with PD-L1 TPS ≥ 50%; however, there was no statistically significant difference among the three levels of PD-L1 TPS groups (*p* = 0.36) (Supplementary Figure S5). Regarding PFS, there were no statistically significant differences between the LIPI 0 and LIPI 1 groups. Almost all PFS were better than the median PFS in the overall analysis group, but not in the PD-L1 TPS 1–49% subgroup of LIPI 1 patients. The median PFS in the PD-L1 TPS ≥ 50% subgroup in the LIPI 2 group was statistically significantly different among the three levels of PD-L1 TPS groups (*p* = 0.034 compared to the PD-L1 TPS < 50% subgroup) and was similar to that of the overall analysis group (8.6 months (95% CI, 4.7–not reached) and 8.6 months (95% CI, 7.3–11.1), respectively) (Supplementary Figure S6).

Patients with poor PNI value and a PD-L1 TPS ≥ 50% and patients with poor LIPI (group 2) and a PD-L1 TPS ≥ 50% had OS and PFS values greater than or equal to the overall median. Based on these results, we performed an analysis of OS and PFS in the PNI 0 group vs. the PNI 1 group and the LIPI 0/1 group vs. the LIPI 2 group, stratified according to whether they had a PD-L1 TPS ≥ 50% or <50%.

**Figure 4:**
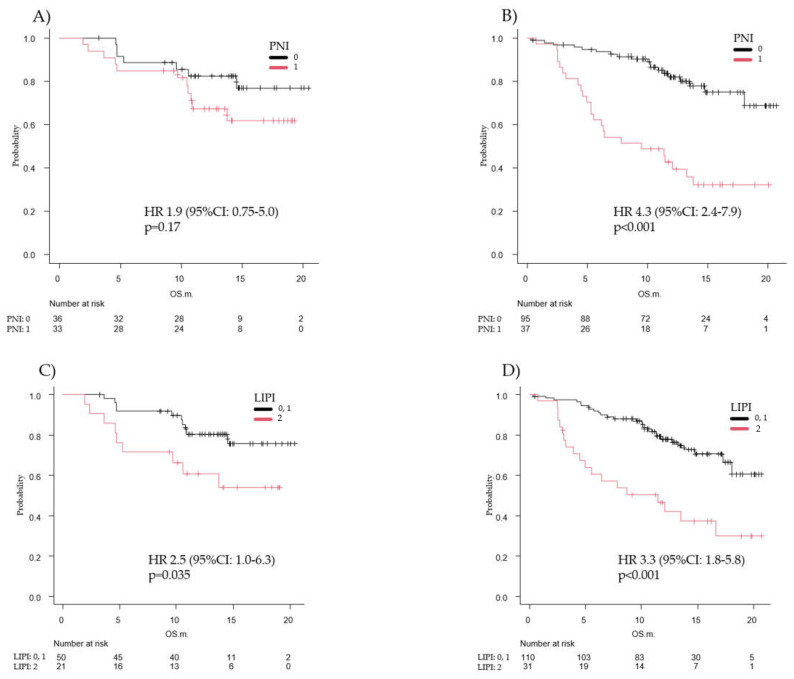
Overall survival analysis in the prognostic nutritional index (PNI) 0 vs. 1 groups and the lung immune prognostic index (LIPI) 0/1 vs. 2 groups stratified by the programmed cell death ligand 1 (PD-L1) tumor proportion score (TPS). Kaplan–Meier estimates of overall survival (OS) in the PD-L1 TPS ≥ 50% group (**A**) and the PD-L1 TPS < 50% group (**B**) according to the PNI. Kaplan–Meier estimates of OS in the PD-L1 TPS ≥ 50% group (**C**) and the PD-L1 TPS < 50% group (**D**) according to the LIPI. The median OS was not reached in the PNI 0 and 1 groups among patients with PD-L1 TPS ≥50% (**A**) (*p* = 0.165). The median OS was not reached in the PNI 0 group and was 9.5 in the PNI 1 group among patients with PD-L1 TPS < 50% (**B**) (*p* < 0.001). The median OS for the LIPI 0/1 and 2 groups was not reached among patients with PD-L1 TPS ≥50% nor among patients with PD-L1 TPS < 50% (**C**) (*p* = 0.035). The median OS was not reached in the LIPI 0/1 group and was 11.5 months in the LIPI 2 group among patients with PD-L1 TPS < 50% (**D**) (*p* < 0.001).

The median OS in the PNI 0 and PNI 1 groups was not reached among patients with PD-L1 TPS ≥ 50% (HR, 1.9; 95% CI, 0.75–5.0; *p* = 0.17). Among patients with PD-L1 TPS < 50%, the median OS was not reached in the PNI 0 group and was 9.5 months in the PNI 1 group (HR, 4.3; 95% CI, 2.4–7.9; *p* < 0.001). Among patients with PD-L1 TPS ≥ 50%, the median OS was not reached in the LIPI 0, LIPI 1, and LIPI 2 groups (HR, 2.5; 95% CI, 1.0–6.3; *p* = 0.035). Among patients with PD-L1 TPS < 50%, OS was 11.5 months (HR, 3.3; 95% CI, 1.8–5.8; *p* < 0.001) (Figure 4).

The median PFS in patients with PNI 0 was 12.6 months in the PD-L1 TPS ≥50% subgroup and 9.6 months in the PD-L1 TPS < 50% subgroup (HR, 1.2; 95% CI, 0.60–2.3; *p* = 0.63). The median PFS in patients with PNI 1 was 11.3 months in the PD-L1 TPS ≥50% subgroup and 4.2 months in the PD-L1 TPS < 50% subgroup (HR, 3.0; 95% CI, 2.0–4.7; *p* < 0.001). The median OS for LIPI 0/1 patients was 15.2 months in the PD-L1 TPS ≥50% subgroup and 8.6 months in the PD-L1 TPS < 50% subgroup (HR, 1.5; 95% CI, 0.77–3.0; *p* = 0.23). The median OS was 9.2 months in the LIPI 2 group and PD-L1 TPS ≥50% subgroup and 3.9 months in the PD-L1 TPS < 50% subgroup (HR, 2.5; 95% CI, 1.6–3.9; *p* < 0.001) (Supplementary Figure S7).

In summary, a pretreatment PNI 1 and a PD-L1 TPS < 50% could be poor prognostic factors for patients with advanced NSCLC. Moreover, a pretreatment LIPI of 2 was a poor prognostic factor, even in patients with a PD-L1 TPS ≥ 50%.

## 3. Discussion

In this retrospective study, we analyzed the clinical data of patients with advanced lung cancer from 12 Japanese institutions, which are all specialized lung cancer treatment hospitals. Hence, these data represent real-world data on patients with advanced NSCLC in Japan who underwent combination therapy with ICIs and chemotherapy.

The outcomes of our study were similar to those of clinical trials, such as the KEYNOTE-189 and KEYNOTE-407 trials, although our analysis group was older compared to those of the clinical trials. The median ages of patients were 65.0 years in the KEYNOTE-189 and -407 trials and 69.0 years in our analysis group [1,2]. As for the sex of our patients, 78.9% were male; however, this proportion was similar to that reported by clinical trials, such as the KEYNOTE-189 and KEYNOTE-407 trials, that consisted of 62.0% and 79.1% male patients, respectively.

In terms of other patient characteristics, 94% had performance status scores of 0 to 1, only 5% had an ECOG-PS score of <2, and 62.9% of patients had adenocarcinoma. In terms of PD-L1 TPS status, the percentages of PD-L1 TPS-negative patients, patients with a PD-L1 TPS of 1–49%, and patients with a PD-L1 TPS of ≥50% were 26.25%, 34.2%, and 30.0%, respectively, which was a similar distribution to that noted in the KEYNOTE-189 trial and the KEYNOTE-407 trial (31.0%, 31.2%, and 32.2%, respectively, in the Pemb combination group in the KEYNOTE-189 trial and 34.2%, 37.1%, and 26.3%, respectively, in the Pemb combination group in the KEYNOTE-407 trial) [2].

Regarding outcomes of the current study, the DCR was 87.3%, the median PFS was 8.6 months, and the median OS was not reached at 11.7 months, which was the median follow-up time. These results were not inferior to those of the clinical trials. For example, in the KEYNOTE-189 trial, the DCR was 84.6%, the median PFS was 8.8 months, and the median OS was not reached at 10.5 months, which was the median follow-up time [1].

Based on this background, we examined the prognosis of patients who received chemoimmunotherapy based on their clinical parameters including pretreatment PNI and LIPI values. Clinical parameters other than PD-L1 TPS, PNI, and LIPI did not correlate with patient survival.

In terms of PNI, a cut-off value of 40 was originally set by Onodera [8], and our calculated cut-off value was 40.35. These values were extremely close, suggesting that our cut-off value was as reliable as that of Onodera’s. When comparing the PNI 0 (good nutrition, ≥40.35) and the PNI 1 (poor nutrition, <40.35) groups, the PNI 0 group tended to have a better DCR than the PNI 1 group, but the difference was not statistically significant. However, the PNI 0 group had a significantly better PFS and OS (both *p*-values were <0.001). Even among patients with a poor PNI value, those with a PD-L1 TPS ≥ 50% had a similar PFS and OS to those in the PNI 0 group. Therefore, PNI could be a potential prognostic predictor of the results of chemoimmunotherapy for patients with advanced NSCLC with PD-L1 TPS < 50%.

Regarding LIPI, the calculation method was fixed, different from PNI. Even so, NLR, which is a factor of LIPI, originally had an unfixed cut-off value. We calculated the ROC curve to observe the discrepancy between the set cut-off value and our result. Our result, 3.74, was higher than the set cut-off value. We confirmed the validity of this score based on the original report on LIPI [12]. In that report, the NLR cut-off value was derived from one large retrospective study [15]. In that study, the NLR calculation resulted in a cut-off value of 3 (HR, 4.10; 95% CI, 3.08–5.46; *p* < 0.001). Our value, 3.74, was within this 95% CI. After confirming that our data did not deviate significantly, we classified patients with NLR using a cut-off value of 3, as specified.

When we observed the DCR in the LIPI 0 (good immune) group, the LIPI 1 (median immune) group, and the LIPI 2 (poor immune) group, the results were significantly better with improving LIPI status. Moreover, for both PFS and OS, the results were significantly better according to the LIPI status. Even in the poor-LIPI group, patients with a PD-L1 TPS ≥ 50% had a similar PFS to that observed for patients in the LIPI good and median immune groups. In contrast to PNI, there was no significant difference in OS. Even so, the Kaplan–Meier curve in the PD-L1 TPS ≥ 50% group was greater than that in the other groups, and the median OS in the PD-L1 TPS ≥ 50% group was not reached. Therefore, in conclusion, these data are immature; however, at this stage, we can hypothesize that LIPI could be a prognostic predictor of the success of chemoimmunotherapy for patients with advanced NSCLC with a PD-L1 TPS < 50%, but whether LIPI could be a real prognostic predictor for the success of chemoimmunotherapy for patients with advanced NSCLC remains controversial. In the original report on LIPI, they concluded that pretreatment LIPI was correlated with worse outcomes of ICI monotherapy, but not of chemotherapy [12]. Moreover, one report, which retrospectively analyzed 114 patients with advanced NSCLC, has concluded that LIPI could not be a prognostic marker of treatment response to ICIs combined with chemotherapy [16]. In contrast, the subgroup analysis of the IMPOWER150 trial analyzed 1148 participants and concluded that LIPI identified subgroups with significantly different survival following first-line Atezo combination therapy initiation for chemotherapy-naive, metastatic non-squamous NSCLC [17].

Immune and nutritional status can also be used to predict cancer cachexia and sarcopenia status. Cancer cachexia is hypothesized to be a poor prognostic factor for advanced lung cancer with or without cytotoxic anticancer chemotherapy [18]. However, few studies have analyzed the association between prognosis in patients with cancer cachexia and ICI therapy for advanced cancer. Only one review on patients with sarcopenia has reported a decrease in the effect of ICI monotherapy in patients with NSCLC [19].

Poor PNI and LIPI, which negatively influenced the prognosis in this study, may reflect cachexia and sarcopenia status. In the present study, the data required for cachexia or sarcopenia diagnoses, such as body weight, were insufficient; thus, analysis of these factors was not possible. Cancer cachexia is a field that has been attracting significant attention in recent years, with anamorelin, the novel anti-cachexia drug, being approved for insurance in Japan in January 2021. Future analyses on these points and prospective studies of these biomarkers are necessary.

This study has some limitations. The fundamental limitations of the study include the small sample size and the retrospective nature of the analysis. We conducted a multicenter trial and accumulated as many patients as possible; however, since this study was retrospective, a larger sample size is ideal. Larger and prospective trials should be conducted to reveal the clinical benefits of pretreatment in PNI and LIPI as predictive markers. Other limitations were as follows. First, since the OS of the good prognostic groups was not reached, the follow-up periods were insufficient to establish significant findings. However, the median follow-up period was similar to those of large-scale clinical trials, such as phase-three trials for combination therapies. Second, we did not mention the adverse effects of the treatment. Since we requested many facilities for cooperation for the larger sample size, we had to keep the types of clinical data simple. Side effects affect the OS, but these will not significantly affect the results of this study. Third, since this study was conducted only in Japan, there was a racial limitation. To show universality that transcends racial difference, global clinical trials are required. Fourth, our cohort was predominantly male; hence, our data cannot be extrapolated to the female population. Future studies should consider a larger sample size with more female patients. Regardless of the abovementioned limitations, this study has strengths in that the sample population in this study comprised real-world data, and there were few selection biases, such as the subgroup data of clinical trials.

## 4. Materials and Methods

We retrospectively investigated patients aged older than 20 years with advanced NSCLC who were treated with chemoimmunotherapy at 12 Japanese institutions between December 2018 and August 2020. This study was approved by the research ethics committee (Permission ID: ERB-C-1803) of the Kyoto Prefectural University of Medicine and other institutions. Informed consent was obtained in the form of opt-out on the website. Patients who rejected the informed consent were excluded. Patients who previously received systemic anticancer therapy for advanced NSCLC were excluded, but patients who received the first anticancer therapy after the recurrence were included. Patients with a driver mutation, such as an EGFR mutation or an anaplastic lymphoma kinase mutation, were excluded.

The following pretreatment parameters were examined and compared: sex, age; BMI; ECOG-PS [20]; smoking status; types of lung cancer histology (squamous or non-squamous); expression levels of PD-L1 TPS (assessed based on the number of PD-L1-positive cells using the 22C-3 antibody); extrathoracic metastases; serum C-reactive protein (CRP) level (cut-off level, 0.5 mg/dL); serum Alb level (cut-off level, 3.5 g/dL); NLR (cut-off level, 3); serum LDH level (cut-off level was the ULN, which was defined according to the limit of each center); LIPI (derived NLR (dNLR) > 3 and LDH greater than the ULN of each center, separated into three groups: good, 0 factors; intermediate, 1 factor; poor, 2 factors); and PNI (10 × Alb (g/dL) + 0.005 × lymph count (/μL)), with the cut-off value decided according to the PNI ROC curve.

The ORR and DCR were evaluated according to the Response Evaluation Criteria in Solid Tumors guidelines 1.1 edition. All *p*-values were two-sided, and *p*-values ≤ 0.05 were considered statistically significant. All statistical analyses were performed using EZR (Saitama Medical Center, Jichi Medical University, Saitama, Japan), which is a graphical user interface for R (The R Foundation for Statistical Computing, Vienna, Austria). More precisely, it is a modified version of the R commander designed to add statistical functions frequently used in biostatistics [21]. For a multivariate analysis (Cox regression analysis), the following parameters were included as confounding factors: sex, age, BMI, ECOG-PS, smoking status, histology, PD-L1 TPS, extra-thoracic metastasis, and serum CRP level. These factors have already been reported as predictive or prognostic factors for lung cancer [6,7]. In these analyses, good and poor prognoses were determined using the significant cut-off value.

## 5. Conclusions

PNI and LIPI could predict survival in patients with advanced NSCLC treated with chemoimmunotherapy, especially in patients with a PD-L1 TPS < 50%. The results of this study may serve as one of the criteria for the proper use of chemoimmunotherapy in the future.

## Figures and Tables

**Table 1:** Patient characteristics.

	Number	(%)
Overall analysis number	237	
Sex		
Male	187	(78.9)
Age at diagnosis		
Median (IQR)	69	(62–73)
≥65	167	(70.5)
≥75	45	(19.0)
Body mass index before treatment		
Median (IQR)	21.5	(19.4–23.3)
<22	135	(57.0)
Smoking status at diagnosis		
Non-smoker	40	(16.9)
Former or current smoker	196	(82.7)
Average Brinkman index	809.8	
Unknown	1	(0.4)
Histology		
Adenocarcinoma	149	(62.9)
Squamous	67	(28.3)
NSCLC—others	21	(8.9)
Molecular alteration other than EGFR or ALK	0	(0.0)
PD-L1 status *		
Negative (<1%)	62	(26.2)
Positive (1–49%)	81	(34.2)
Positive (≥50%)	71	(30.0)
Unknown	23	(9.7)
Performance status (ECOG)		
0	90	(38.0)
1	134	(56.5)
2	10	(4.2)
3	3	(1.3)
4	0	(0.0)
Stage at diagnosis		
recurrent	45	(19.0)
Pretreatment staging		
I or II	9	(3.8)
IIIA	7	(3.0)
Unknown	29	(12.2)
IIIB	13	(5.5)
IIIC	4	(1.7)
More than stage III	1	(0.4)
IV	174	(73.4)
Metastatic sites		
Extrathoracic	134	(56.5)
Regimen		
CBDCA + PEM + Pemb	124	(52.3)
CBDCA + PTX/nab-PTX + Pemb	83	(35.0)
CBDCA + PTX + Atezo ± Bev	25	(10.5)
CBDCA + PEM + Atezo	5	(2.1)
Response rate		
ORR	140	(59.1)
Complete response	15	(6.3)
Partial response	125	(52.7)
Stable disease	67	(28.3)
Progression	17	(7.2)
NA	13	(5.5)
Disease control rate	207	(87.3)
Lym		
Median (/μL) (IQR)	1360	(1029–1827)
dNLR		
Median (IQR)	3.4	(2.4–5.3)
>3	143	(60.3)
LDH>ULN	78	(32.9)
Alb		
Median (IQR)	3.6	(3.1–4.0)
PNI		
Median (IQR)	42.5	(37.3–47.9)
LIPI		
0	73	(30.8)
1	107	(45.1)
2	57	(24.1)

Note: Patients receiving combination therapy with immune checkpoint inhibitor and cytotoxic anticancer chemotherapy. Footnote: * By immunohistochemistry, 22C-3; IQR, interquartile range; NSCLC, non-small cell lung cancer; EGFR, epidermal growth factor receptor; ALK, anaplastic lymphoma kinase; PD-L1, programmed cell death ligand 1; ECOG, Eastern Cooperative Oncology Group; CBDCA, carboplatin; PEM, pemetrexed; PTX, paclitaxel; Pemb, pembrolizumab; Atezo, atezolizumab; Bev, bevacizumab; ORR, overall response rate; NA, not assessable; Lym, lymphocyte; dNLR, derived neutrophil-to-lymphocyte ratio; LDH, lactate dehydrogenase; ULN, upper limit of normal; Alb, albumin; PNI, prognostic nutritional index; LIPI, lung immune prognostic index.

**Table 2:** Univariate and multivariate analyses for overall survival in the overall analysis group.

Features		Univariate Analysis for OS				Multivariate Analysis for OS			
		HR	95% CI		*p*-Value	HR	95% CI		*p*-Value
Sex	Male/Female	0.85	0.48–1.50	1.50	0.58	0.75	0.32	1.78	0.52
Age	≥75/<75	2.12	1.28	3.50	<0.001	2.39	1.29	4.43	0.01
BMI	≥22/>22	0.65	0.41	1.04	0.07	0.64	0.37	1.12	0.12
PS	>2/0,1	2.35	1.13	4.90	0.02	0.76	0.22	2.64	0.67
Smoking status	Yes/No	1.76	0.88	3.53	0.11	1.24	0.47	3.30	0.66
Sq	Sq/non-Sq	1.33	0.83	2.13	0.23	0.92	0.51	1.66	0.78
PD-L1	≥50/<50	0.75	0.44	1.28	0.30	0.53	0.30	0.95	0.03
Extra-thoracic metastasis	Yes/No	1.16	0.73	1.82	0.53	0.88	0.50	1.52	0.64
CRP	>0.5/≤0.5	1.89	1.16	3.08	0.01	0.93	0.47	1.81	0.82
Alb	<3.5/≥3.5	3.15	1.95	5.10	<0.001				
NLR	>3.0/≤3.0	2.71	1.58	4.64	<0.001				
LDH>ULN	>ULN/≤ULN	2.07	1.33	3.23	<0.001				
LIPI (0 or 1) vs. 2	2/(0 or 1)	2.40	1.52	3.79	<0.001	2.75	1.48	5.11	<0.001
PNI	<40.35/≥40.35	3.00	1.88	4.77	<0.001	2.38	1.23	4.62	0.01

Note: Univariate and multivariate analyses for overall survival in the overall analysis group performed using Cox regression analysis revealed that age <75 years (hazard ratio [HR], 2.39; *p* = 0.01), lung immune prognostic index (LIPI) score 0 or 1 (HR, 2.75; *p* < 0.001), and prognostic nutritional index (PNI) ≥40.35 (HR, 2.38; *p* = 0.01) were favorable independent prognostic factors. Footnote: OS, overall survival; HR, hazard ratio; CI, confidence interval; BMI, body mass index; PS, performance status; Sq, squamous cell carcinoma; PD-L1, programmed cell death ligand 1; CRP, C-reactive protein; Alb, albumin; NLR, neutrophil-to-lymphocyte ratio; LDH, lactate dehydrogenase; ULN, upper limit of normal; LIPI, lung immune prognostic index; PNI, prognostic nutritional index.

## Data Availability

The data that support the findings of this study are available from the corresponding author, Junji Uchino, upon reasonable request.

## Supplementary Materials

The following supporting information can be downloaded at: https://www.mdpi.com/article/10.3390/diagnostics12020423/s1, Figure S1: Study flowchart. Among the 253 patients with advanced non-small cell lung carcinoma who received combination therapy with immune checkpoint inhibitors and cytotoxic anticancer chemotherapy, 16 (6.3%) were excluded owing to driver mutations and previous administration of chemoimmunotherapy. The final number of patients for analysis was 237. NSCLC: non-small cell lung carcinoma, KPUM: Kyoto Prefectural University of Medicine; Figure S2. Overall survival (OS) and progression-free survival (PFS) in the overall analysis group according to the programmed cell death ligand 1 (PD-L1) tumor proportion score (TPS). Kaplan–Meier estimates of OS (A) and PFS (B) in the overall analysis group according to the PD-L1 TPS. Green line, PD-L1 TPS ≥ 50%; red line, PD-L1 TPS 1–49%; and black line, PD-L1 TPS < 1%. There were no significant differences among the groups (OS: *p* = 0.51 and PFS: *p* = 0.054); Figure S3. Overall survival in the prognostic nutritional index (PNI) 0 and 1 groups according to the programmed cell death ligand 1 (PD-L1) tumor proportion score (TPS). Kaplan–Meier estimates of overall survival (OS) in patients with a PNI score of 0 (good-nutrition group) (A) and PNI score 1 (poor-nutrition group) (B) according to the PD-L1 TPS. Green line, PD-L1 TPS ≥ 50%; red line, PD-L1 TPS 1–49%; and black line, PD-L1 TPS < 1%. In the PNI 0 group (A), there was no significant difference in OS based on PD-L1 TPS (*p* = 0.87). In the PNI 1 group (B), survival improved significantly with increasing PD-L1 TPS (*p* = 0.045); Figure S4. Progression-free survival (PFS) in the prognostic nutritional index (PNI) 0 and 1 groups according to the programmed cell death ligand 1 (PD-L1) tumor proportion score (TPS). Kaplan–Meier estimates of PFS in the PNI score 0 (good-nutrition) group (A) and the PNI score 1 (poor-nutrition) group (B) according to the PD-L1 TPS. Green line, PD-L1 TPS ≥ 50%; red line, PD-L1 TPS 1–49%; and black line, PD-L1 TPS < 1%. In the PNI 0 group, there was no significant difference in PFS based on PD-L1 TPS (*p* = 0.76). In the PNI 1 group, the survival improved significantly with increasing PD-L1 TPS (*p* < 0.001); Figure S5. Overall survival (OS) in the lung immune prognostic index 0, 1, or 2 groups according to the programmed cell death ligand 1 (PD-L1) tumor proportion score (TPS). Kaplan–Meier estimates of OS in the lung immune prognostic index (LIPI) score 0 (good immune status) group (A), LIPI score 1 (median immune status) group (B), and the LIPI score 2 (poor immune status) group (C) according to the PD-L1 TPS. Green line, PD-L1 TPS ≥ 50%; red line, PD-L1 TPS 1–49%; and black line, PD-L1 TPS < 1%. There was no significant difference in any of these groups (*p* = 0.32, *p* = 0.66, and *p* = 0.36, respectively); Figure S6. Progression-free survival (PFS) in the lung immune prognostic index (LIPI) 0, 1, or 2 groups according to the programmed cell death ligand 1 (PD-L1) tumor proportion score (TPS). Kaplan–Meier estimates of PFS in the LIPI score 0 (good immune status) group (A), the LIPI score 1 (median immune status) group (B), and the LIPI score 2 (poor immune status) group (C) according to the PD-L1 TPS. Green line, PD-L1 TPS ≥ 50%; red line, PD-L1 TPS 1–49%; and black line, PD-L1 TPS < 1%. There were no significant differences in the LIPI 0 and LIPI 1 groups (*p* = 0.81 and *p* = 0.093, respectively). In the LIPI 2 group, the PFS significantly improved with increasing PD-L1 TPS (*p* = 0.034); Figure S7. Progression-free survival analysis in the prognostic nutritional index (PNI) 0 and 1 groups and the lung immune prognostic index (LIPI) 0/1 and 2 groups according to the programmed cell death ligand 1 (PD-L1) tumor proportion score (TPS). Kaplan–Meier estimates of progression-free survival (PFS) in the PD-L1 TPS ≥50% group (A) and the PD-L1 TPS < 50% group (B) according to the PNI. Kaplan–Meier estimates of PFS in the PD-L1 TPS ≥ 50% group (C) and the PD-L1 TPS < 50% group (D) according to the LIPI. The median OS was 12.6 months in the PNI 0 group and 9.6 months in the PNI 1 group among patients with PD-L1 TPS ≥50% (A) (hazard ratio (HR,) 1.2; *p* = 0.63), and 11.3 in the PNI 0 group and 4.2 in the PNI 1 group among patients with PD-L1 TPS < 50% (B) (HR, 3.0; *p* < 0.001). The median OS was 15.2 months in the LIPI 0/1 group and 8.6 in the LIPI 2 group among patients with PD-L1 TPS ≥50% (C) (HR, 1.5; *p* = 0.23), and 9.2 months in the LIPI 0/1 group and 3.9 months in the LIPI 2 group among patients with PD-L1 TPS < 50% (D) (HR, 2.5; *p* < 0.001); Table S1: Univariate and multivariate analyses for progression-free survival in the overall analysis group. Univariate and multivariate analyses for progression-free survival in the overall analysis group performed using Cox regression analysis revealed that a lung immune prognostic index (LIPI) score of 0 or 1 (hazard ratio (HR), 2.01; *p* < 0.001) and a prognostic nutritional index (PNI) ≥ 40.35 (HR, 1.72; *p* = 0.03) were favorable independent prognostic factors. The PD-L1 tumor proportion score was also significantly associated with PFS in both the univariate and multivariate analyses (HR, 0.42; *p* < 0.001).
