# Supplementary material for: Prognostic Nutritional Index and Lung Immune Prognostic Index as Prognostic Predictors for Combination Therapies of Immune Checkpoint Inhibitors and Cytotoxic Anticancer Chemotherapy for Patients with Advanced Non-Small Cell Lung Cancer
Source: Diagnostics (Basel). 2022 Feb 6;12(2):423. doi: 10.3390/diagnostics12020423 (PMC8870759; doi:10.3390/diagnostics12020423)
Supplement: Supplementary file 1 [file diagnostics-12-00423-s001.zip › diagnostics-1552261-supplementary.pdf]

Supplementary Materials

Table S1. Univariate and multivariate analyses for progression-free survival in the overall analysis group. Univariate and multivariate analyses for progression-free survival in the overall analysis group performed using Cox regression analysis revealed that a lung immune prognostic index (LIPI) score of 0 or 1 (hazard ratio (HR), 2.01; $p < 0.001$) and a prognostic nutritional index (PNI) ≥ 40.35 (HR, 1.72; $p = 0.03$) were favorable independent prognostic factors. The PD-L1 tumor proportion score was also significantly associated with PFS in both the univariate and multivariate analyses (HR, 0.42; $p < 0.001$).

**Features**		**Univariate Analysis for PFS**					**Multivariate Analysis for PFS**				
		**HR**	**95% CI**			***p* value**	**HR**	**95% CI**			***p*-value**
Sex	Male/Female	1.23	0.84	-	1.79	0.29	1.34	0.79		2.27	0.28
Age	≥75/<75	1.53	1.03	-	2.26	0.03	1.39	0.88	-	2.19	0.16
BMI	≥22/>22	0.68	0.49	-	0.95	0.02	0.75	0.50	-	1.11	0.15
PS	≥2/0,1	1.37	0.70	-	2.69	0.36	0.58	0.20	-	1.65	0.31
Smoking status	Yes/No	1.17	0.75	-	1.83	0.49	1.20	0.67	-	2.15	0.54
Sq	Sq/non-Sq	1.44	1.02	-	2.03	0.04	1.36	0.90	-	2.05	0.15
PD-L1	≥50/<50	0.63	0.43	-	0.92	0.02	0.42	0.27	-	0.65	<0.001
Extra-thoracic metastasis	Yes/No	1.12	0.81	-	1.56	0.48	0.92	0.63	-	1.35	0.67
CRP	>0.5/≤0.5	1.60	1.14	-	2.25	0.01	1.55	0.98	-	2.44	0.06
Alb	<3.5/≥3.5	1.96	1.41	-	2.73	0.00				-	
NLR	>3.0/≤3.0	1.40	1.00	-	1.96	0.05				-	
LDH>ULN	>ULN/≤ULN	1.53	1.10	-	2.12	0.01				-	
PNI	<40.35/≥40.35	1.87	1.34	-	2.62	0.00	2.01	1.28	-	3.15	<0.001
LIPI (0 or 1) vs. 2	2/(0 or 1)	1.75	1.23	-	2.49	0.00	1.72	1.07	-	2.79	0.03

Footnote: PFS, progression-free survival; HR, hazard ratio; CI, confidence interval; BMI, body mass index; PS, performance status; Sq, squamous cell carcinoma; PD-L1, programmed cell death ligand 1; CRP, C-reactive protein; Alb, albumin; NLR, neutrophil-to-lymphocyte ratio; LDH, lactate dehydrogenase; ULN, upper limit of normal; PNI, prognostic nutritional index; LIPI, lung immune prognostic index.

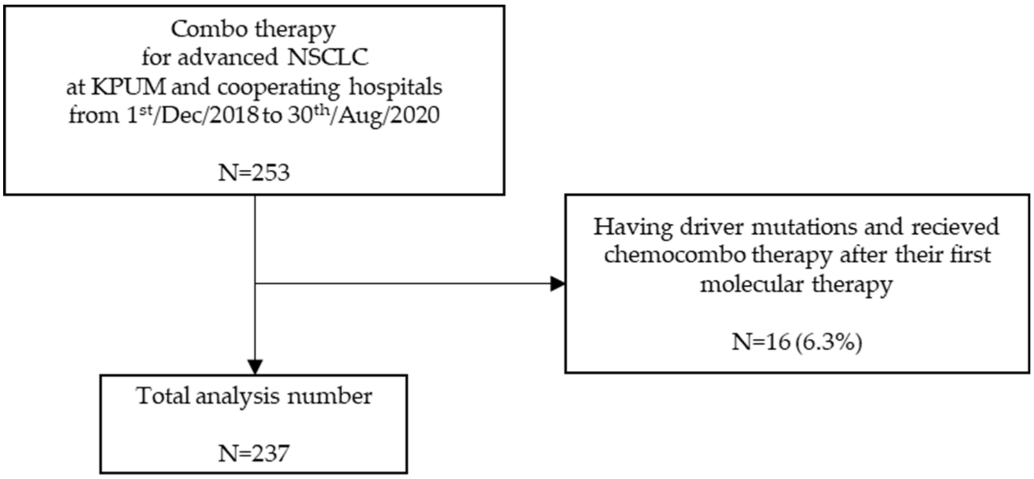

Figure S1. Study flowchart. Among the 253 patients with advanced non-small cell lung carcinoma who received combination therapy with immune checkpoint inhibitors and cytotoxic anticancer chemotherapy, 16 (6.3%) were excluded owing to driver mutations and previous administration of chemoimmunotherapy. The final number of patients for analysis was 237. NSCLC: non-small cell lung carcinoma, KPUM: Kyoto Prefectural University of Medicine.

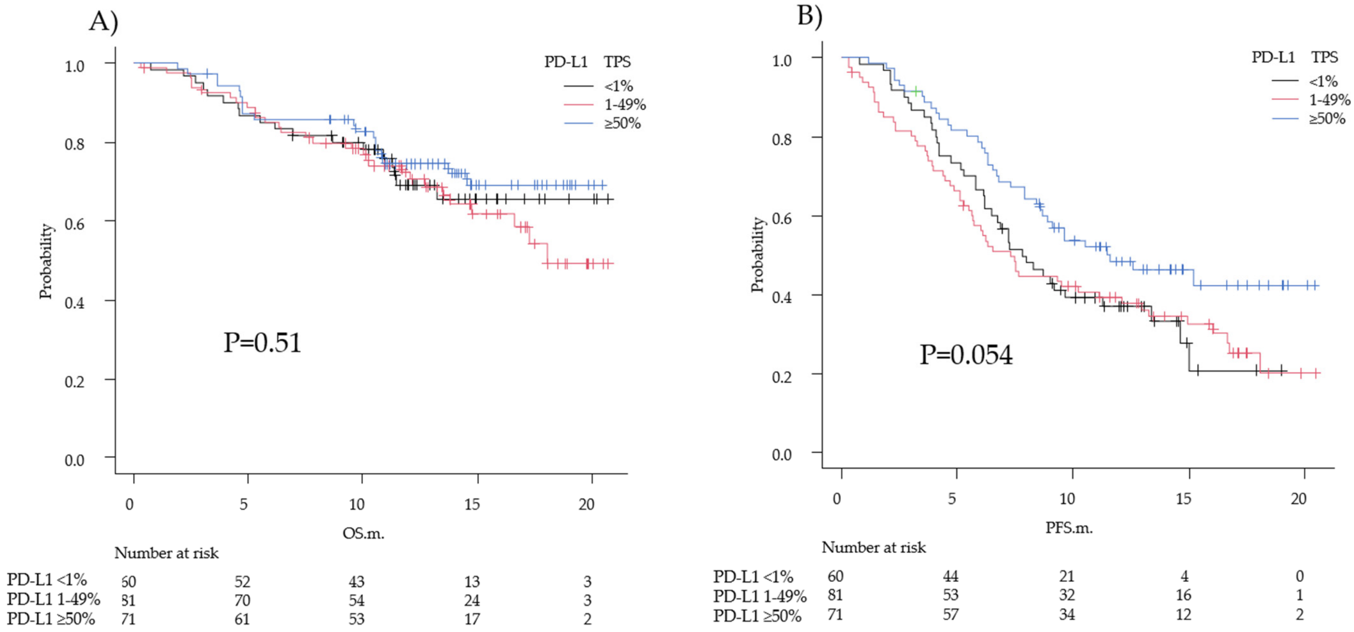

Figure S2. Overall survival (OS) and progression-free survival (PFS) in the overall analysis group according to the programmed cell death ligand 1 (PD-L1) tumor proportion score (TPS). Kaplan–Meier estimates of OS (A) and PFS (B) in the overall analysis group according to the PD-L1 TPS. Green line, PD-L1 TPS ≥ 50%; red line, PD-L1 TPS 1–49%; and black line, PD-L1 TPS < 1%. There were no significant differences among the groups (OS: $p = 0.51$ and PFS: $p = 0.054$).

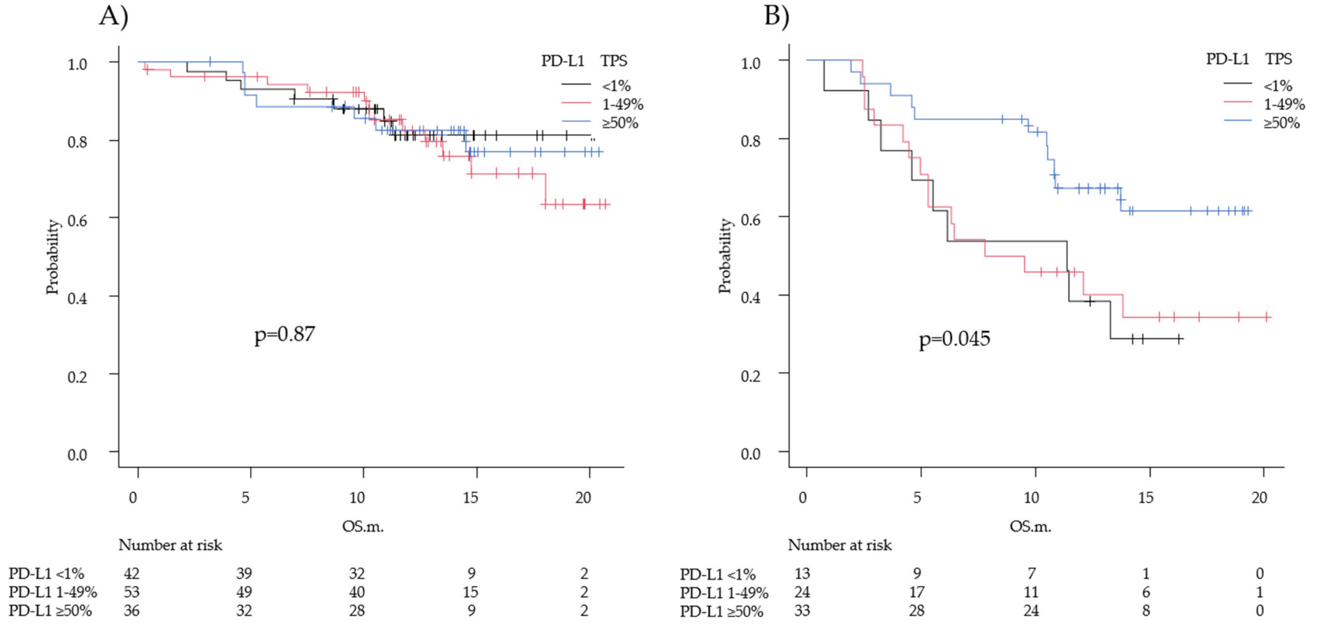

Figure S3. Overall survival in the prognostic nutritional index (PNI) 0 and 1 groups according to the programmed cell death ligand 1 (PD-L1) tumor proportion score (TPS). Kaplan–Meier estimates of overall survival (OS) in patients with a PNI score of 0 (good-nutrition group) (**A**) and PNI score 1 (poor-nutrition group) (**B**) according to the PD-L1 TPS. Green line, PD-L1 TPS ≥ 50%; red line, PD-L1 TPS 1–49%; and black line, PD-L1 TPS < 1%. In the PNI 0 group (**A**), there was no significant difference in OS based on PD-L1 TPS ($p = 0.87$). In the PNI 1 group (**B**), survival improved significantly with increasing PD-L1 TPS ($p = 0.045$).

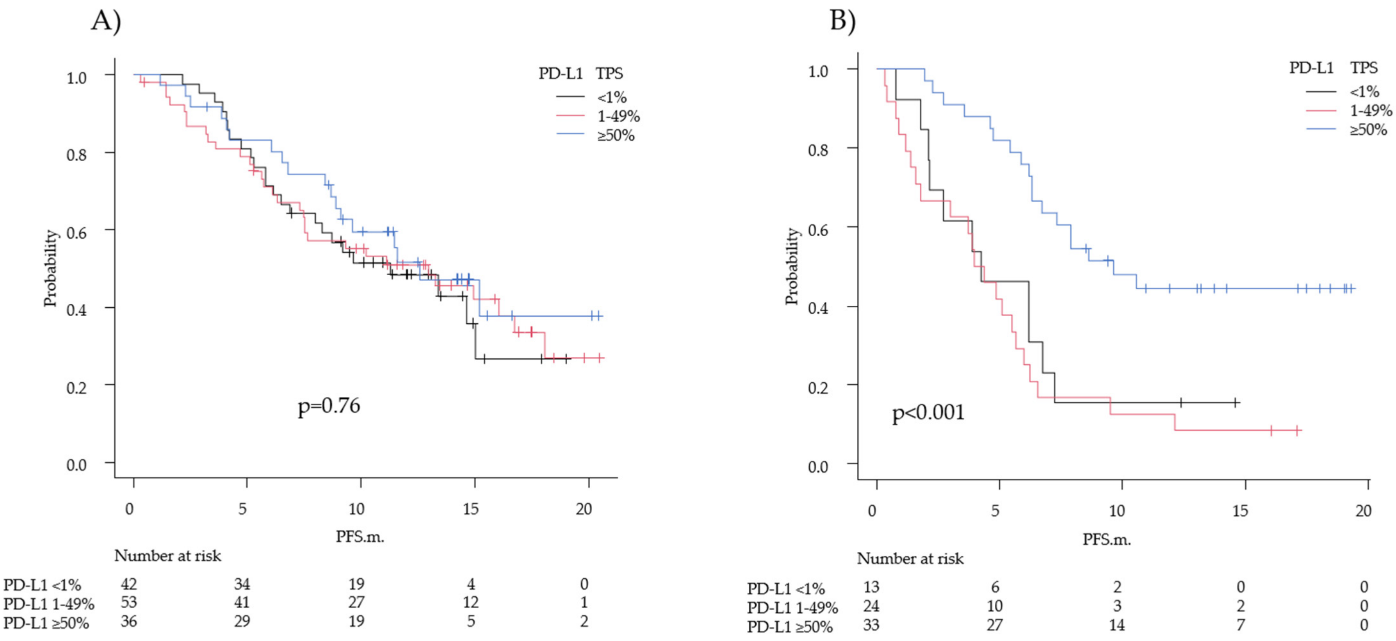

Figure S4. Progression-free survival (PFS) in the prognostic nutritional index (PNI) 0 and 1 groups according to the programmed cell death ligand 1 (PD-L1) tumor proportion score (TPS). Kaplan–Meier estimates of PFS in the PNI score 0 (good-nutrition) group (**A**) and the PNI score 1 (poor-nutrition) group (**B**) according to the PD-L1 TPS. Green line, PD-L1 TPS ≥ 50%; red line, PD-L1 TPS 1–49%; and black line, PD-L1 TPS < 1%. In the PNI 0 group, there was no significant difference in PFS based on PD-L1 TPS (p = 0.76). In the PNI 1 group, the survival improved significantly with increasing PD-L1 TPS (p < 0.001).

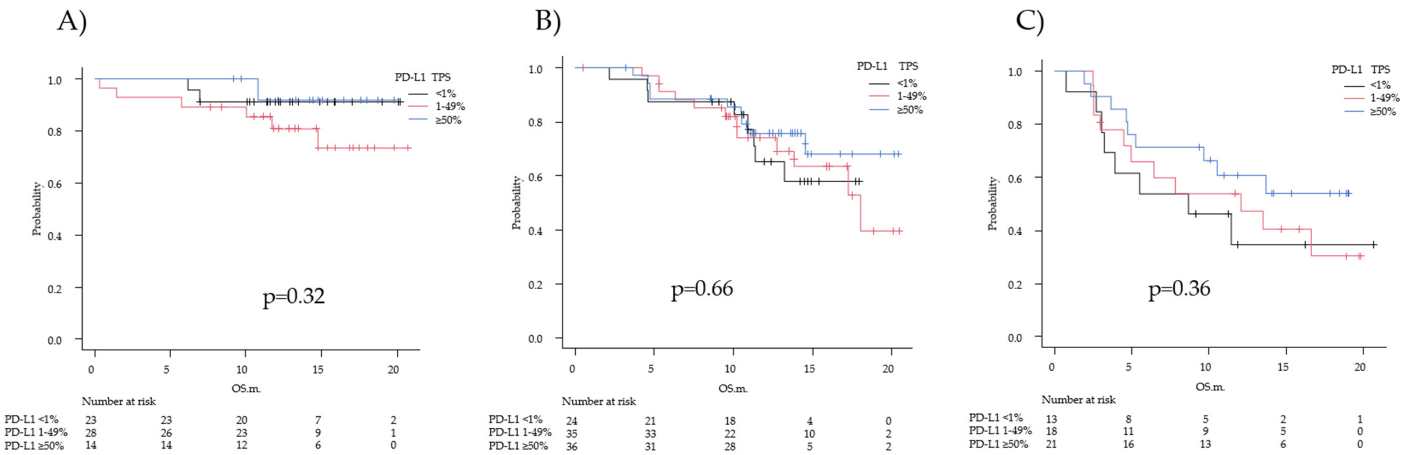

Figure S5. Overall survival (OS) in the lung immune prognostic index 0, 1, or 2 groups according to the programmed cell death ligand 1 (PD-L1) tumor proportion score (TPS). Kaplan–Meier estimates of OS in the lung immune prognostic index (LIPI) score 0 (good immune status) group (**A**), LIPI score 1 (median immune status) group (**B**), and the LIPI score 2 (poor immune status) group (**C**) according to the PD-L1 TPS. Green line, PD-L1 TPS ≥ 50%; red line, PD-L1 TPS 1–49%; and black line, PD-L1 TPS < 1%. There was no significant difference in any of these groups (p = 0.32, p = 0.66, and p = 0.36, respectively).

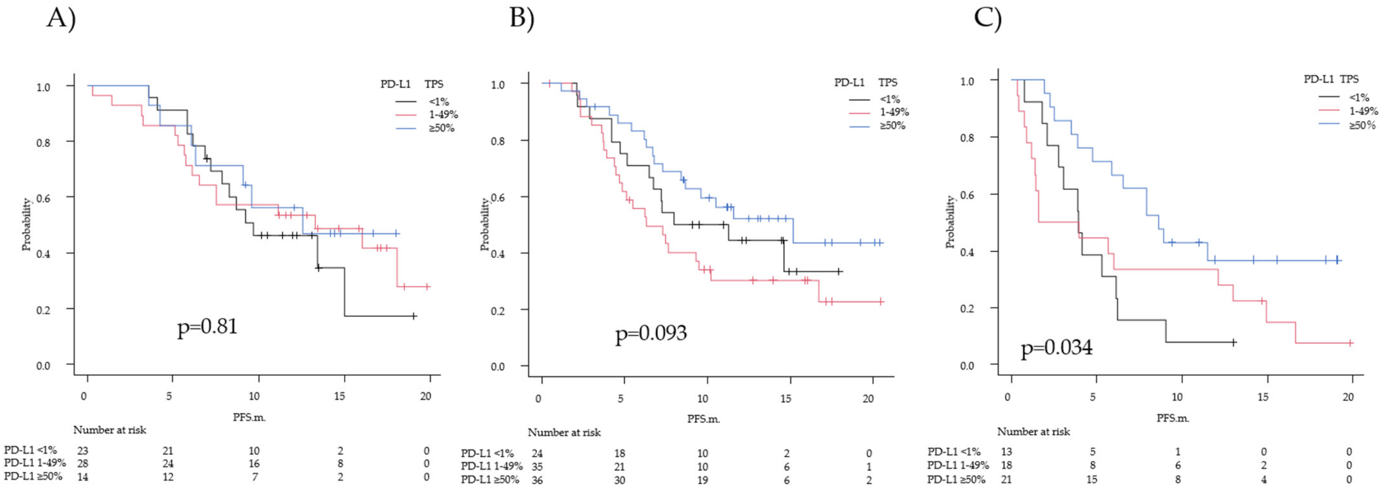

Figure S6. Progression-free survival (PFS) in the lung immune prognostic index (LIPI) 0, 1, or 2 groups according to the programmed cell death ligand 1 (PD-L1) tumor proportion score (TPS). Kaplan–Meier estimates of PFS in the LIPI score 0 (good immune status) group (**A**), the LIPI score 1 (median immune status) group (**B**), and the LIPI score 2 (poor immune status) group (**C**) according to the PD-L1 TPS. Green line, PD-L1 TPS ≥ 50%; red line, PD-L1 TPS 1–49%; and black line, PD-L1 TPS < 1%. There were no significant differences in the LIPI 0 and LIPI 1 groups (p = 0.81 and p = 0.093, respectively). In the LIPI 2 group, the PFS significantly improved with increasing PD-L1 TPS (p = 0.034).

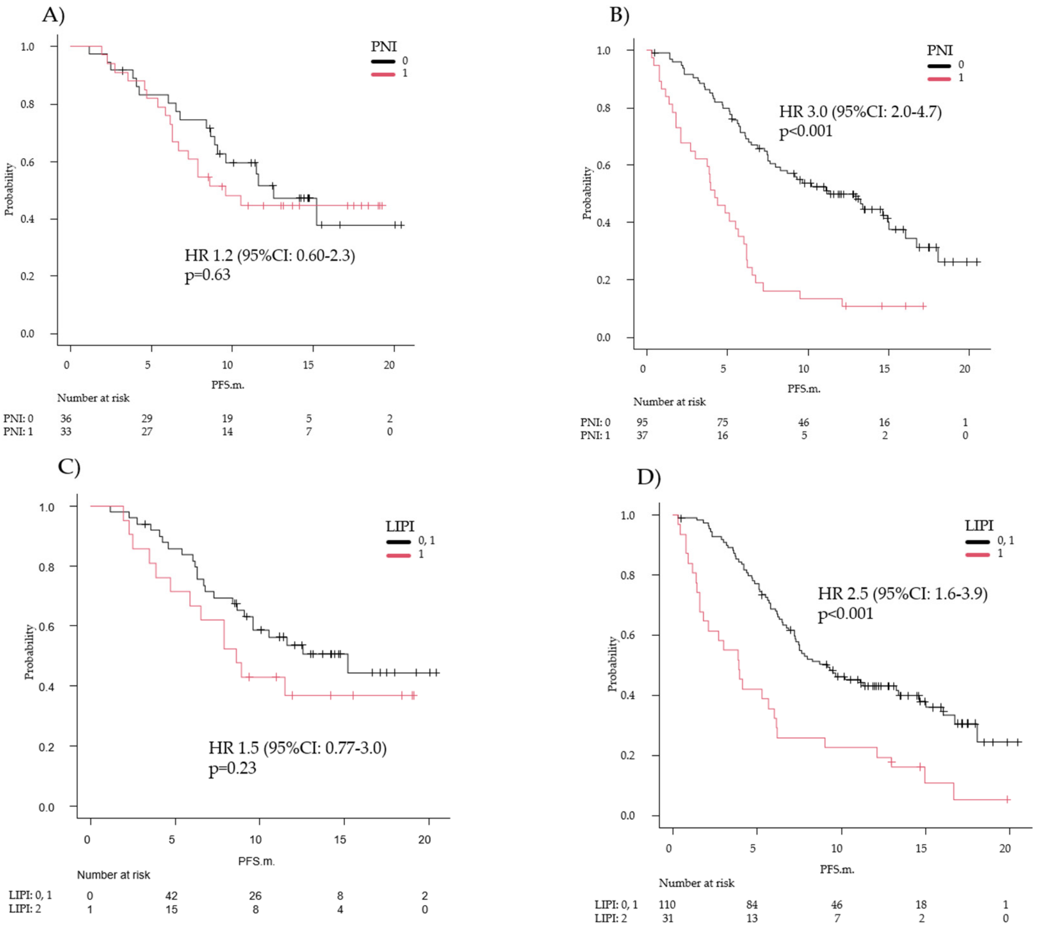

Figure S7. Progression-free survival analysis in the prognostic nutritional index (PNI) 0 and 1 groups and the lung immune prognostic index (LIPI) 0/1 and 2 groups according to the programmed cell death ligand 1 (PD-L1) tumor proportion score (TPS). Kaplan–Meier estimates of progression-free survival (PFS) in the PD-L1 TPS ≥50% group (**A**) and the PD-L1 TPS < 50% group (**B**) according to the PNI. Kaplan–Meier estimates of PFS in the PD-L1 TPS ≥ 50% group (**C**) and the PD-L1 TPS < 50% group (**D**) according to the LIPI. The median OS was 12.6 months in the PNI 0 group and 9.6 months in the PNI 1 group among patients with PD-L1 TPS ≥50% (**A**) (hazard ratio (HR,) 1.2; p = 0.63), and 11.3 in the PNI 0 group and 4.2 in the PNI 1 group among patients with PD-L1 TPS < 50% (**B**) (HR, 3.0; p < 0.001). The median OS was 15.2 months in the LIPI 0/1 group and 8.6 in

the LIPI 2 group among patients with PD-L1 TPS ≥50% (**C**) (HR, 1.5; $p = 0.23$), and 9.2 months in the LIPI 0/1 group and 3.9 months in the LIPI 2 group among patients with PD-L1 TPS < 50% (**D**) (HR, 2.5; $p < 0.001$).
